# Peptide-Based Nanoparticle for Tumor Therapy

**DOI:** 10.3390/biomedicines13061415

**Published:** 2025-06-09

**Authors:** Phonpilas Thongpon, Menghuan Tang, Zhaoqing Cong

**Affiliations:** 1 Department of Biochemistry & Molecular Medicine, University of California Davis, Sacramento, CA 95817, USA; phonpilasth@gmail.com (P.T.); tmhtang@ucdavis.edu (M.T.); 2 South China Hospital, Medical School, Shenzhen University, Shenzhen 518116, China

**Keywords:** peptide nanoparticles, cancer therapy, self-assembly, nanomedicine

## Abstract

Cancer treatment continues to face significant challenges due to the limitations of conventional therapies, including non-specific toxicity, poor bioavailability, and drug resistance. Nanotechnology, particularly peptide-based nanoparticles (NPs), is increasingly recognized as a valuable strategy to address these obstacles. Peptides provide a versatile platform offering high biocompatibility, specificity, biodegradability, and minimal immunogenicity, making them ideal for targeted cancer therapies. This review comprehensively examines recent advancements in peptide-based nanoparticle systems, highlighting the mechanisms driving peptide self-assembly, such as amphiphilicity, non-covalent interactions, and metal coordination. It distinguishes between non-bioactive peptide nanoparticles, which primarily serve as drug carriers, and bioactive peptide nanoparticles, which integrate targeting peptides, cell-penetrating peptides (CPPs), and therapeutic peptides to enhance specificity, internalization, and anticancer efficacy. Emphasis is placed on innovative designs that exploit active targeting, stimuli-responsive release, and immunomodulatory strategies to maximize therapeutic outcomes while minimizing side effects. Despite promising preclinical outcomes, the clinical translation of peptide nanoparticles struggles with challenges involving stability, delivery efficiency, scalability, regulatory compliance, and manufacturing complexity. The review concludes by outlining future directions, emphasizing personalized nanomedicine, combination therapies, and advanced peptide engineering as crucial pathways toward successful clinical implementation.

## 1. Introduction

### 1.1. Context: Limitations of Conventional Cancer Therapies

Cancer remains a major global health challenge and a leading cause of mortality worldwide [1]. Standard treatment modalities, including surgery, radiotherapy, and chemotherapy, form the cornerstone of current oncology practice [2]. However, these conventional approaches often face significant limitations. Chemotherapy and radiotherapy, while effective in killing rapidly dividing cells, lack specificity for malignant cells, frequently leading to considerable collateral damage to healthy tissues and resulting in severe systemic toxicities [3]. Common side effects such as myelosuppression, neurotoxicity, cardiotoxicity, hair loss, and gastrointestinal distress can compromise the patient’s quality of life and may necessitate treatment discontinuation or dose reduction, potentially impairing therapeutic outcomes [4]. Furthermore, the intrinsic or acquired resistance of cancer cells to therapeutic agents poses a major obstacle, often leading to treatment failure and disease relapse [3]. The high global cancer burden, with projections indicating millions of new cases annually [5], underscores the pressing need for safer and more effective therapeutic strategies. This necessitates the development of targeted therapies capable of selectively acting upon tumor cells or modulating the tumor microenvironment (TME), the complex ecosystem of cells, blood vessels, and molecules surrounding the tumor, thereby enhancing treatment efficacy while minimizing harm to normal tissues [3].

### 1.2. Nanotechnology in Oncology: Nanoparticles as Delivery Platforms

Nanotechnology has emerged as a transformative force in medicine, offering innovative solutions to long-standing challenges in drug delivery, particularly in oncology [3].

#### 1.2.1. Nanoparticles (NPs)

Nanoparticles, defined as materials typically sized between 1 and 100 nanometers (though sometimes up to 1000 nm in nanomedicine contexts), serve as versatile platforms for carrying therapeutic and/or diagnostic agents [3]. Their application in cancer therapy stems from several key advantages they provide compared to conventional drug formulations [3]. NPs can enhance the solubility and stability of poorly water-soluble drugs (e.g., paclitaxel) [2], protect therapeutic cargo from enzymatic degradation in the bloodstream [2], enhance drug bioavailability [2], and enable controlled or sustained release kinetics, potentially maintaining drug concentrations within the therapeutic window for longer periods and reducing dosing frequency [6]. Common NP platforms explored in oncology include liposomes, polymeric nanoparticles, dendrimers, micelles, inorganic nanoparticles (e.g., gold, silica, iron oxide), and protein-based nanoparticles (e.g., albumin, ferritin) [7].

#### 1.2.2. Passive Tumor Targeting

A significant feature exploited by NPs in cancer therapy is the enhanced permeability and retention (EPR) effect [3]. Many solid tumors exhibit aberrant vasculature characterized by leaky endothelial junctions (with pore sizes ranging from 100 nm to 2 µm) and impaired lymphatic drainage [8]. Nanoparticles within an appropriate size range (typically 10–100 nm, though optimal size varies) can extravasate through these leaky vessels and become trapped within the tumor interstitium, leading to passive accumulation at the tumor site relative to healthy tissues [3]. However, the EPR effect is known to be highly heterogeneous, varying significantly between tumor types, stages, locations, and even within the same tumor, and often results in relatively low overall NP accumulation (reported as less than 1% of the administered dose in many cases) [9,10,11].

#### 1.2.3. Active Tumor-Targeting Strategies

This recognition of EPR’s limitations has spurred the development of active targeting strategies, where NP surfaces are functionalized with ligands (e.g., antibodies, aptamers, peptides) that specifically target receptors that are overexpressed on cancer cells or components of the TME [7]. This active targeting aims to enhance tumor-specific accumulation and cellular uptake, further improving therapeutic precision.

While nanoparticles offer distinct advantages as delivery platforms, their full therapeutic potential in oncology is often significantly enhanced when combined with functional biomolecules, such as peptides, which will be discussed in the following section.

### 1.3. Peptides in Nanomedicine: Properties and Potential

Peptides, defined as short chains of amino acids (typically 2–50 residues) linked by peptide bonds, represent a distinct and highly versatile class of biomolecules with burgeoning applications in medicine, particularly in cancer diagnosis and therapy [12]. Their appeal stems from a unique combination of advantageous properties. Peptides often exhibit high specificity and selectivity for their biological targets, such as cell surface receptors or enzymes, enabling precise molecular interactions [12]. Derived from natural building blocks, they generally possess excellent biocompatibility and biodegradability, breaking down into amino acids and minimizing long-term accumulation concerns [2]. Compared to larger biologics like antibodies, peptides typically exhibit lower immunogenicity and toxicity, particularly to organs like the bone marrow and liver [13]. Their smaller size facilitates synthesis, chemical modification, and potentially better penetration into dense tissues like solid tumors [12].

Despite their many advantages, peptides face inherent limitations that have historically hindered their therapeutic development. Their susceptibility to enzymatic degradation by proteases in the bloodstream and tissues leads to poor in vivo stability and short biological half-lives [4]. Rapid renal clearance further contributes to their short circulation times [4]. While often less immunogenic than proteins, some peptides can still elicit immune responses [13]. Additionally, their binding affinity for targets may be lower than that of antibodies [14]. These challenges necessitate strategies to enhance peptide stability and pharmacokinetic profiles for effective therapeutic application.

### 1.4. Peptide-Based Nanoparticles: A Synergistic Approach

Integrating peptides with nanotechnology to create peptide-based nanoparticles offers a synergistic approach, particularly for targeted cancer therapy, by harnessing the strengths of both components while mitigating their individual weaknesses [1]. These are sophisticated systems where nanoparticles serve as protective carriers, enhancing the peptide’s stability against degradation and prolonging its circulation time, while the peptide imparts specific functionalities such as targeting, cell penetration, or direct therapeutic action [4]. This synergy is central to the field, moving beyond simple drug encapsulation towards highly engineered, multi-functional therapeutic agents.

**Figure 1:**
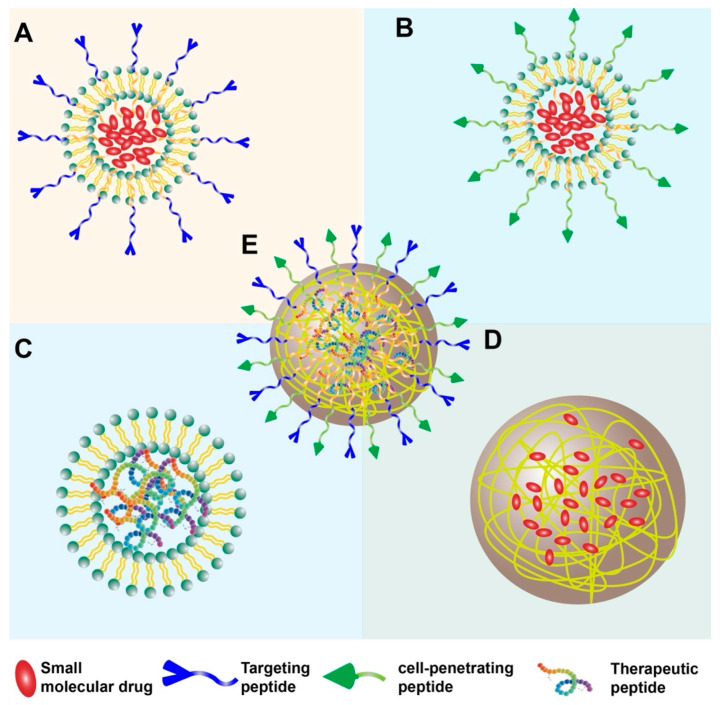
Overview of peptide nanoparticle strategies for cancer therapy. (**A**) Nanoparticle core (e.g., polymer, lipid) with targeting peptides conjugated to the surface. (**B**) Nanoparticle core with cell-penetrating peptides (CPPs) conjugated to the surface. (**C**) Nanoparticle encapsulating therapeutic peptides as cargo. (**D**) Nanostructure formed by the self-assembly of peptides (e.g., micelles, nanofibers) encapsulating a drug. (**E**) Multifunctional nanoparticle combining several elements (e.g., targeting peptide + CPP + drug payload).

Peptides can be incorporated into nanoparticle systems in numerous ways. Peptides can be conjugated to the surface of pre-formed nanoparticles (made of polymers, lipids, inorganic materials, etc.) to serve as targeting ligands that recognize specific markers on cancer cells or the TME (bioactive role) [4] (Figure 1A). Cell-penetrating peptides (CPPs) can be added to facilitate the uptake of nanoparticles and their cargo into target cells [15] (Figure 1B). Furthermore, peptides themselves can be the therapeutic payload, with intrinsic anticancer or immunomodulatory activity, and are delivered more effectively via nanoparticle formulation [16] (Figure 1C). Alternatively, they can act as fundamental building blocks, self-assembling into defined nanostructures that encapsulate therapeutic payloads (structural role) [1] (Figure 1D). Peptides can also be designed to make multifunctional nanoparticles, combining several elements (e.g., targeting peptide + CPP + drug payload) (Figure 1E). Despite promising preclinical results, the clinical translation of these systems faces hurdles, including stability, delivery efficiency, and manufacturing challenges [1].

A particularly dynamic area is the application of peptide-based nanoparticles in cancer immunotherapy [17]. The success of immunotherapy has revolutionized cancer treatment, but response rates remain limited in many patients [17]. Peptide-based nanoparticles offer promising tools to enhance immunotherapeutic strategies by serving as platforms for peptide vaccines (delivering tumor antigens and adjuvants) [18], delivering immunomodulatory peptides (e.g., checkpoint inhibitors) [18], or targeting immunosuppressive cells within the TME [18]. This integration aligns the field of peptide nanomedicine with the broader paradigm shift towards immune-based cancer treatments.

### 1.5. Review Scope and Structure

This review aims to provide a comprehensive overview of the recent advancements (with a focus on the literature published since 2020) in the field of peptide-based nanoparticles for tumor therapy. It will delve into the fundamental principles governing their formation, explore the different types of peptide nanoparticles based on the peptide’s role (structural vs. bioactive), and discuss their diverse applications in targeting and treating cancer. Specifically, the following sections will cover (2) the driving forces behind peptide self-assembly into nanostructures and methods to control it; (3) nanoparticles based on non-bioactive peptides used primarily as carriers; (4) nanoparticles incorporating bioactive peptides for targeting, cell penetration, and direct therapeutic effects (including pro-apoptotic and immunomodulatory actions); and (5) a concluding discussion summarizing the current status, significant challenges (including manufacturing and regulatory aspects), and future prospects of this rapidly evolving field.

Having outlined the rationale and scope for employing peptide-based nanoparticles in tumor therapy, a fundamental understanding of how these nanostructures form is essential. Therefore, the following section will delve into the key driving forces that govern peptide self-assembly.

## 2. Driving Forces for Self-Assembly

### 2.1. Introduction to Peptide Self-Assembly

Peptide self-assembly is a fundamental process wherein individual peptide molecules spontaneously organize into well-defined, ordered supramolecular structures at the nanoscale [2]. This phenomenon, driven by a delicate balance of various non-covalent interactions, leads to the formation of diverse morphologies, including nanofibers, nanotubes, nanoribbons, nanovesicles, micelles, and hydrogels [19]. The ability of peptides, even short sequences, to encode the necessary molecular information for forming these complex architectures makes them attractive building blocks for nanomaterials [20].

The significance of peptide self-assembly in nanomedicine, particularly for cancer therapy, lies in its ability to generate biocompatible and biodegradable nanomaterials with tunable properties [2]. These self-assembled nanostructures can serve as efficient carriers for therapeutic agents, offering high loading capacity for both hydrophobic and hydrophilic drugs, protecting the cargo from degradation, and enabling controlled release profiles [2]. The self-assembly process itself is highly sensitive to both the intrinsic characteristics of the peptide—such as its amino acid sequence, length, charge distribution, and hydrophobicity—and the external environmental conditions, including pH, temperature, ionic strength, solvent composition, and the presence of guest molecules or metal ions [2]. This sensitivity provides a powerful avenue for rationally designing and controlling the self-assembly process to yield nanostructures with desired morphologies, stabilities, and functionalities tailored for specific biomedical applications. Understanding the driving forces governing this process is therefore crucial for the development of effective peptide-based nanotherapeutics.

### 2.2. Amphiphilic Structure

A primary driver for the self-assembly of many peptides, particularly in aqueous environments, is their amphiphilic nature [21]. Amphiphilic peptides possess distinct regions with differing affinities for water: a hydrophobic (water-repelling) segment and a hydrophilic (water-attracting) segment. When placed in an aqueous solution, these molecules spontaneously organize to minimize the thermodynamically unfavorable interactions between the hydrophobic domains and water molecules [21]. Typically, the hydrophobic segments aggregate together, forming a core that is shielded from the aqueous environment, while the hydrophilic segments remain exposed to the water, forming the outer surface or corona of the resulting nanostructure [21]. This process leads to the formation of various supramolecular architectures such as micelles (spherical structures with a hydrophobic core and hydrophilic shell), vesicles or liposomes (spherical structures with an aqueous core enclosed by a bilayer membrane), and nanofibers or nanotubes (elongated structures formed by the stacking of peptide molecules) [19]. Peptide amphiphiles (PAs), which often consist of a hydrophobic alkyl tail conjugated to a hydrophilic peptide sequence, are classic examples of molecules designed to exploit amphiphilicity for controlled self-assembly into nanofibers and other structures relevant for drug delivery and tissue engineering [1]. The balance between the hydrophobic and hydrophilic portions significantly influences the critical aggregation concentration and the morphology of the self-assembled structures.

### 2.3. Key Non-Covalent Interactions

**Figure 2:**
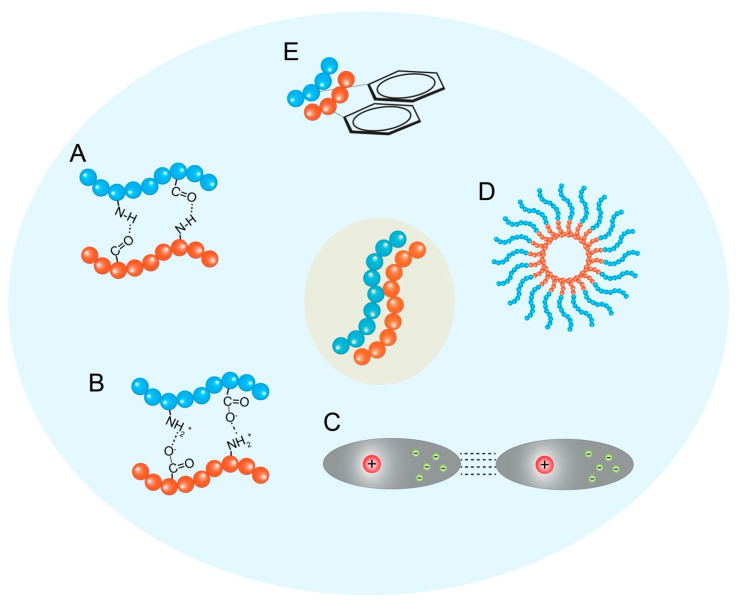
Driving forces and control of peptide self-assembly. The key non-covalent interactions, (**A**) hydrogen bonding, (**B**) electrostatic interactions, (**C**) van der Waals forces, (**D**) hydrophobic interactions, and (**E**) π–π stacking, drive peptide self-assembly. It could also visually represent how factors like pH, temperature, or peptide sequence modification can influence the resulting nanostructure morphology (e.g., micelles vs. nanofibers vs. vesicles).

While amphiphilicity provides a strong initial impetus for aggregation, the stability, specific morphology, and internal order of self-assembled peptide nanostructures are governed by a complex interplay of multiple, relatively weak non-covalent interactions acting cooperatively [2]. The final assembled state represents a thermodynamic minimum energy state achieved through the synergistic effects of these forces [22]. Key interactions include:

Hydrogen Bonding: Hydrogen bonds are directional interactions crucial for defining the secondary structure of peptides (e.g., β-sheets, α-helices), which often serve as the fundamental motifs that template the hierarchical self-assembly process [2] (Figure 2A). Hydrogen bonds form between the amide hydrogen (donor) and carbonyl oxygen (acceptor) atoms in the peptide backbone, as well as potentially involving amino acid side chains [23]. In β-sheet-rich structures like amyloid fibrils or nanofibers, extensive intermolecular hydrogen bonding perpendicular to the peptide chain direction provides significant structural stability [23]. The selectivity and directionality of hydrogen bonds play a critical role in the formation and stabilization of specific peptide conformations and ordered nanostructures [2].

Electrostatic Interactions: Interactions between charged amino acid residues (e.g., Lys, Arg, His vs. Asp, Glu) play a significant role, particularly for peptides containing ionizable groups [2] (Figure 2B). Attractive forces between oppositely charged groups can promote assembly and stabilize structures, while repulsive forces between like charges can hinder aggregation or influence the final morphology. These interactions are highly dependent on the pH and ionic strength of the surrounding medium, providing a means to control assembly via environmental triggers [23]. Electrostatic interactions are also important for the encapsulation and retention of charged drug molecules within peptide nanocarriers [23].

Van der Waals Interactions: These are weak, short-range attractive forces arising from temporary fluctuations in electron distribution [21] (Figure 2C). While individually weak, the cumulative effect of numerous van der Waals interactions between closely packed atoms within the self-assembled structure contributes significantly to the overall stability and cohesion of the aggregate [24].

Hydrophobic Interactions: As discussed under amphiphilicity, the tendency of nonpolar amino acid side chains (and potentially conjugated hydrophobic moieties) to aggregate and minimize contact with water is a major thermodynamic driving force for self-assembly in aqueous solutions (Figure 2D) [2]. While powerful in inducing aggregation, hydrophobic interactions are generally considered less directional than hydrogen bonds or π–π stacking [7].

π–π Stacking Interactions: These interactions occur between the electron clouds of aromatic amino acid side chains, such as those of phenylalanine (Phe), tyrosine (Tyr), tryptophan (Trp), and histidine (His) [3]. π–π stacking is directional and contributes significantly to the stability and ordered packing within self-assembled structures, particularly for peptides rich in aromatic residues or those conjugated with bulky aromatic groups (e.g., Fmoc or fluorenylmethyloxycarbonyl, naphthalene, pyrene) [2]. These interactions can induce the directional growth of assemblies and often lead to more stable structures in aqueous solutions due to the poor solubility of aromatic groups [23] (Figure 2E). The specific geometry (e.g., face-to-face, edge-to-face) of π–π stacking influences the final architecture and potential electronic properties of the assembly [25].

The delicate balance and competition among these various non-covalent forces ultimately dictate the energy landscape, kinetic pathways, and thermodynamic stability of the self-assembly process, leading to the diverse range of observed nanostructures [21].

### 2.4. Metal Coordination Interactions

Beyond the canonical non-covalent forces, metal coordination has emerged as a distinct and powerful driving force for directing peptide self-assembly, offering unique opportunities for structural control and functionalization [2]. Inspired by the intricate structures and functions of natural metalloproteins (proteins that contain a metal ion cofactor), researchers utilize metal–ligand interactions to orchestrate the formation of well-defined peptide-based nanomaterials [26].

The fundamental mechanism involves the formation of coordination bonds between specific metal ions and electron-donating atoms within the peptide sequence, which act as ligands [26]. Certain amino acid side chains are particularly adept at coordinating metal ions, notably histidine (via its imidazole nitrogen atoms), cysteine (via its thiol group), and aspartate/glutamate (via carboxylate oxygens) [2]. Additionally, backbone amide nitrogens and carbonyl oxygens, as well as terminal amino and carboxyl groups, can participate in coordination [26]. To expand the possibilities, artificial metal-binding ligands such as pyridine, bipyridine, terpyridine, or crown ethers can be synthetically incorporated into the peptide sequence [26].

A wide range of metal ions have been employed, including transition metals (e.g., Zn^2+^, Cu^2+^, Ni^2+^, Co^2+^, Fe^2+^/^3+^, Ag^+^) and alkaline earth metals (e.g., Mg^2+^, Ca^2+^). Each metal ion exhibits characteristic coordination numbers and preferred geometries (e.g., tetrahedral, square planar, octahedral), which, combined with the number and placement of coordinating residues within the peptide, dictate the resulting supramolecular architecture [26]. This allows for the programmed assembly of diverse nanostructures, ranging from simple nanoparticles and nanofibers to more complex, discrete architectures like cages, capsules, or prisms [2]. For instance, the coordination of Zn(II) with cyclic dihistidine peptides resulted in fluorescent nanoparticles capable of encapsulating epirubicin [2], while specific arrangements of metal clusters and peptide ligands yielded highly ordered Ni_6_L_4_ capsules, Ni₉L_6_ prisms, and Ni₁₈L₁_2_ octahedron cages [27].

A key feature distinguishing metal coordination from other non-covalent interactions is the combination of strength, directionality, and often, reversibility [26]. Coordination bonds generally have a bonding strength between that of hydrogen bonds/van der Waals forces and covalent bonds, providing stability while allowing for dynamic behavior [26]. The well-defined geometry of coordination bonds imparts directionality to the assembly process, enabling the construction of highly ordered structures [26]. Furthermore, the reversibility of many metal–ligand interactions allows the assemblies to become sensitive to external stimuli such as changes in redox potential, temperature, or light, or the interaction of competing ligands, making them suitable for applications requiring controlled disassembly or release [26]. Metal coordination can also introduce specific functionalities derived from the metal ion itself, such as catalytic activity, redox properties, magnetic properties (e.g., Mn^2+^ for MRI), or antimicrobial effects (e.g., Ag^+^) [26].

These unique characteristics make metal-coordinated peptide assemblies highly relevant for biomedical applications, including stimuli-responsive drug delivery, biological imaging using the metal ion as a contrast agent, antimicrobial hydrogels, and scaffolds for tissue engineering that leverage the biological roles of metal ions [27]. Thus, metal coordination represents a sophisticated tool for peptide self-assembly, providing an additional layer of control over structure and function beyond the traditional non-covalent interactions.

### 2.5. Controlling Self-Assembly

The ability to precisely control the self-assembly process is paramount for designing peptide nanoparticles with the desired characteristics for therapeutic applications [19]. Control can be exerted by manipulating both the intrinsic properties of the peptide building blocks and the external environmental conditions [2].

Intrinsic Peptide Factors: The amino acid sequence is the primary determinant of self-assembly behavior [2]. Factors like peptide length, the balance and distribution of hydrophobic and hydrophilic residues (amphiphilicity), the presence and position of charged amino acids, aromatic residues (for π-π stacking), and specific secondary structure propensities (e.g., β-sheet-forming sequences) all dictate the type and stability of the resulting nanostructure [2]. Introducing modifications like unnatural amino acids, cyclization, or conjugation with other molecules (e.g., polymers, lipids, bulky aromatic groups like Fmoc) provides further handles for tuning assembly [2].

External Environmental Factors: The self-assembly process is highly sensitive to the surrounding environment [2]. Key parameters include the following:

- pH: Affects the protonation state of ionizable amino acid side chains (e.g., His, Asp, Glu, Lys, Arg), altering electrostatic interactions and hydrogen bonding patterns [2]. This allows for pH-triggered assembly or disassembly, relevant for drug release in acidic tumor microenvironments [19].
- Temperature: Influences hydrophobic interactions and the kinetics of assembly. Some systems, like ELPs, exhibit specific inverse temperature transitions [2].
- Ionic Strength: Modulates electrostatic interactions by screening charges, which can either promote or inhibit assembly depending on the peptide sequence [19].
- Solvent Composition: The polarity and type of solvent significantly impact hydrophobic interactions and peptide conformation [2].
- Peptide Concentration: Self-assembly typically occurs above a critical aggregation concentration [21].
- Presence of Co-solutes/Interfaces: Interactions with guest molecules (drugs), metal ions, or surfaces can template or direct the assembly process [19].

By carefully designing the peptide sequence and controlling these external factors, researchers can guide the self-assembly towards specific morphologies (nanofibers, vesicles, micelles, etc.), control nanoparticle size, stability, and responsiveness, ultimately tailoring the nanocarrier for optimal performance in cancer therapy [19].

With a foundational understanding of the principles dictating peptide self-assembly and its control, we can now explore the different categories of resulting nanoparticle systems. This review will first examine nanoparticles where peptides primarily serve a structural or carrier role, as detailed in the next section on non-bioactive peptide-based nanoparticles.

## 3. Non-Bioactive Peptide-Based Nanoparticles for Tumor Therapy

### 3.1. Introduction

In the context of peptide-based nanoparticles for cancer therapy, one major category comprises systems where the peptide component primarily serves a structural or carrier function, rather than imparting specific biological activity like targeting or direct cytotoxicity. These are often referred to as “non-bioactive” peptide-based nanoparticles, although the term signifies the intended primary role of the peptide component in the design, rather than an absolute lack of any biological interaction. The main goal of these systems is to leverage the advantageous physicochemical properties conferred by the peptide structure—such as self-assembly capability, biocompatibility, biodegradability, and drug encapsulation potential—to improve the delivery of conventional chemotherapeutics or other therapeutic agents [28]. These nanoparticles typically rely on passive accumulation in tumors via the EPR effect [3] or may incorporate features for stimuli-responsive release, but the peptide itself is not designed to actively guide the nanoparticle to a specific molecular target or kill cancer cells directly. This section explores prominent examples of such systems, including polypeptide nanoparticles, teledendrimer peptide nanoparticles, and elastin-like polypeptide nanoparticles, highlighting how their peptide-derived structures are exploited for improved drug delivery. Even without active targeting peptides, these systems represent an important step beyond free drug administration by improving pharmacokinetics and potentially mitigating systemic toxicity, and they often serve as platforms upon which targeting or other bioactive functionalities can later be built.

### 3.2. Polypeptide Nanoparticles

**Figure 3:**
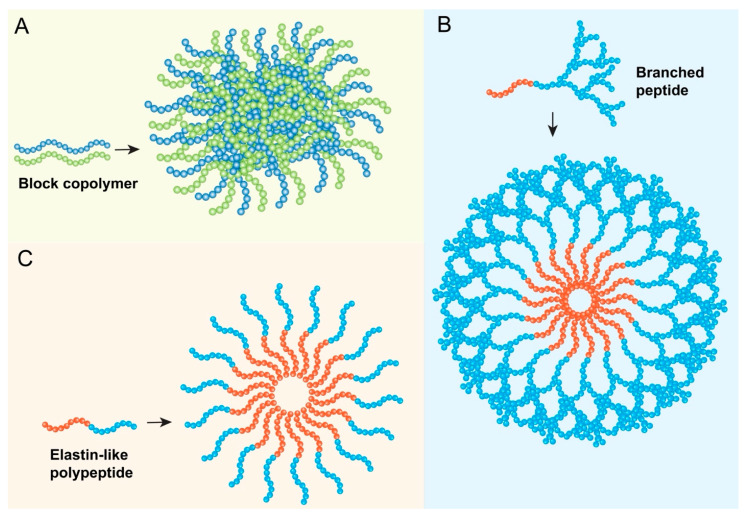
Examples of non-bioactive peptide nanoparticle architectures. (**A**) Polypeptide nanoparticle formulated from longer chains of amino acids. (**B**) Peptide dendrimer nanoparticles formulated from branched polypeptides. (**C**) Elastin-like polypeptides (ELPs) system.

Polypeptide nanoparticles encompass a broad range of nanostructures formulated from longer chains of amino acids, which can be either synthetic homopolymers, block copolymers containing polypeptide segments, or recombinant proteins [28] (Figure 3A). These materials can self-assemble or be processed into various morphologies, including micelles, nanospheres, nanogels, polymersomes (vesicles formed from amphiphilic block copolymers), and conjugates [28]. The peptide backbone provides biocompatibility and biodegradability, while the specific amino acid composition and polymer architecture dictate the nanoparticle’s properties, such as its hydrophobicity/hydrophilicity balance, drug-loading capacity, stability, and release characteristics. While sometimes used interchangeably with self-assembling short peptides or peptide–polymer conjugates in the literature, the term “polypeptide nanoparticles” often implies systems where longer, pre-formed or recombinantly produced polypeptide chains form the core structural basis of the nanoparticle, distinct from nanoparticles that are merely surface-functionalized with short peptides [1].

A primary application of polypeptide nanoparticles in cancer therapy is as carriers for conventional chemotherapeutic drugs, particularly those with poor water solubility or unfavorable pharmacokinetics [3]. By encapsulating drugs like doxorubicin, paclitaxel, or cisplatin within the nanoparticle core or matrix, these systems can enhance drug solubility, protect the drug from premature leaking, enhance its circulation time in the bloodstream, and promote its accumulation in lesion tissues through the EPR effect [3]. This can lead to improved therapeutic efficacy and potentially reduced systemic toxicity compared to the administration of the free drug [3]. For instance, polymeric micelles formed from block copolymers containing polypeptide segments, such as NC-6004 (a cisplatin-incorporating micelle based on PEG-poly(glutamic acid)), and polypeptide–drug conjugates like AP5280 (HPMA copolymer–doxorubicin conjugate with a GFLG linker) have progressed to clinical trials, aiming to improve the therapeutic index of established chemotherapies [29].

Strategies such as PEGylation (attaching polyethylene glycol chains) are commonly employed to further enhance the stability of polypeptide nanoparticles, reduce their uptake by the reticuloendothelial system (RES), prolong their circulation half-life, and improve tumor accumulation [3]. Furthermore, polypeptide nanoparticles hold potential for overcoming multidrug resistance (MDR) in cancer cells [3]. By delivering drugs via endocytosis, nanoparticles may bypass the P-glycoprotein (P-gp) and other efflux pumps responsible for expelling drugs from resistant cells [30]. Additionally, the high payload capacity of nanoparticles allows for the co-delivery of chemotherapeutic agents along with MDR inhibitors (e.g., COX-2 inhibitors, siRNA-targeting efflux pumps) within a single carrier, offering a synergistic approach to combat resistance [30]. While primarily structural, the polypeptide component provides the essential framework for these advanced delivery capabilities.

### 3.3. Teledendrimer Peptide Nanoparticles

Dendrimers, characterized by their highly branched, three-dimensional architecture, represent a unique class of synthetic macromolecules and precisely defined architecture emanating from a central core [5] (Figure 3B). They are synthesized layer-by-layer, with each layer referred to as a generation (G0, G1, G2, etc.), resulting in a globular structure with a high density of functional groups on the periphery and potential internal cavities [31]. “Teledendrimer” typically refers to structures where dendritic wedges (dendrons) are attached to a linear polymer backbone or other core structures. Peptide dendrimers incorporate peptide units either as the branching monomers, as surface functionalities, or conjugated to a non-peptide dendritic scaffold [32]. Poly(amidoamine) (PAMAM) dendrimers are a commonly studied type used in biomedical applications [33].

In the context of non-bioactive carriers, peptide dendrimers offer several advantages for drug delivery. Their well-defined structure and size allow for predictable behavior [34]. The numerous peripheral functional groups provide ample sites for drug conjugation or surface modification (e.g., PEGylation for improved biocompatibility), while the internal voids or hydrophobic domains within the branched structure can encapsulate guest molecules, leading to high drug-loading capacities [31]. Peptide dendrimers have been explored as carriers for various anticancer drugs, including doxorubicin and gemcitabine [32]. Dendrimers can improve the water solubility and bioavailability of hydrophobic drugs due to their internal hydrophobic environment [32].

A key feature that can be incorporated into peptide dendrimer systems is stimuli-responsiveness. By conjugating drugs to the dendrimer periphery via peptide linkers that are susceptible to cleavage by enzymes overexpressed in the tumor microenvironment (e.g., cathepsin B cleaving the Gly-Phe-Leu-Gly sequence), controlled drug release specifically at the tumor site can be achieved [32]. For example, a mPEGylated peptide dendrimer–doxorubicin conjugate using the GFLG linker self-assembled into nanoparticles and demonstrated enzyme-responsive drug release and significant antitumor activity in a breast tumor model with reduced systemic toxicity [32].

However, challenges associated with dendrimers include their relatively complex multi-step synthesis and potential cytotoxicity, particularly for higher-generation cationic dendrimers, which can interact non-specifically with cell membranes [34]. Surface modifications, such as PEGylation or conjugation with biocompatible polymers like carboxymethylchitosan, are often necessary to mitigate toxicity and improve in vivo performance [32]. Dendrimers have also been extensively used as surface modifiers for inorganic nanoparticles (e.g., gold, selenium), where the dendrimer layer enhances stability, drug/gene-loading, and provides a platform for attaching targeting ligands, effectively bridging the gap towards active targeting systems [33]. For instance, PAMAM dendrimers have been conjugated to gold nanoparticles (AuNPs) via Au-S or Au-N bonds or encapsulated within them, serving as carriers for drugs like DOX or genes (siRNA, DNA), often incorporating targeting ligands like folic acid or antibodies [33]. Similarly, PAMAM-functionalized selenium nanoparticles (SeNPs) have been used for the targeted co-delivery of siRNA and cisplatin [33].

### 3.4. Elastin-like Polypeptide (ELP) Nanoparticles

Elastin-like polypeptides (ELPs) are a fascinating class of genetically engineered biopolymers inspired by the repetitive sequences found in human tropoelastin, the precursor to elastin [35]. They typically consist of repeating pentapeptide units, most commonly Val-Pro-Gly-Xaa-Gly (VPGXG), where ‘Xaa’ represents a “guest” amino acid residue that can be any amino acid except proline, and ‘n’ denotes the number of repeats [36]. This structure imparts several highly desirable properties for biomedical applications: excellent biocompatibility and biodegradability (breaking down into natural amino acids), low immunogenicity (particularly for humanized sequences), and precise control over molecular weight and sequence, which is achievable through recombinant production techniques [35].

The most distinctive characteristic of ELPs is their stimuli-responsive behavior, specifically an inverse temperature transition [35]. Below a specific transition temperature (Tt), ELPs are soluble in aqueous solutions as unimers. However, upon heating above the Tt, they undergo a reversible phase transition, desolvating and aggregating to form structures such as coacervates (dense liquid phases formed by phase separation) or nanoparticles [37]. This Tt is precisely tunable by altering the hydrophobicity of the guest residue (Xaa), the number of repeats (n, i.e., the molecular weight), and the ELP concentration [38]. This thermo-responsiveness allows for the simple, non-chromatographic purification of recombinantly produced ELPs via temperature cycling [39] and forms the basis for several drug delivery strategies.

In cancer therapy, ELPs primarily serve as non-bioactive carriers or structural components (Figure 3C), exploiting the following unique properties:

Thermally Targeted Delivery: ELPs can be designed with a Tt slightly above physiological temperature (e.g., 40–42 °C). When conjugated to a drug and administered systemically, the ELP–drug conjugate remains soluble in circulation. Applying mild, localized hyperthermia specifically to the tumor site can raise the temperature above the Tt, causing the ELP to aggregate within the tumor vasculature or interstitium, thereby concentrating the drug payload at the target site and facilitating its release [37]. ELP–doxorubicin conjugates have demonstrated efficacy using this approach [37].

Enhanced Pharmacokinetics and Passive Targeting: High-molecular-weight ELPs (e.g., >70 kDa) exhibit prolonged circulation times due to the reduced renal clearance [40]. This extended half-life increases the probability of accumulation in tumors via the EPR effect [40]. ELP conjugation can thus improve the biodistribution and tumor-targeting of attached drugs or proteins [40]. Studies show that higher-molecular-weight ELPs (e.g., 160 kDa) exhibit greater tumor accumulation compared to lower-molecular-weight ones (e.g., 25 kDa) [41].

Drug/Biologic Conjugation: ELPs serve as versatile carriers for various therapeutic cargoes. Small-molecule drugs (e.g., doxorubicin, paclitaxel) can be chemically conjugated, often via stimuli-responsive linkers (e.g., acid-labile hydrazone for lysosomal release) [37]. Therapeutic peptides and proteins can be fused to ELPs at the genetic level, allowing for the recombinant production of well-defined fusion proteins [35]. ELPs have also been used to deliver photosensitizers for photodynamic therapy [35]. Recent work includes ELP-based nanoparticles designed to deliver miRNA, incorporating targeting (AP1 peptide) and penetrating (Tat peptide) motifs alongside the ELP structure for stability and ligand presentation [42].

Nanoparticle Formation/Stabilization: ELPs that phase separate can self-assemble into nanoparticles (micelles, coacervates) above their Tt, encapsulating drugs during the assembly process [37]. Alternatively, ELPs designed to remain soluble at physiological temperatures can be used as hydrophilic coronas to sterically stabilize other types of nanoparticles (e.g., liposomes, inorganic NPs), improving their colloidal stability and pharmacokinetic properties [36].

The combination of biocompatibility, precise genetic control over structure, tunable thermo-responsiveness, and versatile conjugation chemistry makes ELPs a highly promising platform within the realm of non-bioactive peptide-based nanoparticles for cancer therapy, bridging the gap between simple passive delivery and more sophisticated, stimuli-responsive systems. Their potential role in overcoming drug resistance, similar to other polypeptide carriers, by altering delivery mechanisms is also an area of interest [40].

### 3.5. Peptide Hydrogels as Drug Delivery Systems

Peptide hydrogels, formed through the self-assembly of short synthetic or rationally designed peptides into three-dimensional nanofibrous networks, have emerged as versatile platforms for localized and sustained drug delivery in cancer therapy [43]. These hydrogels are typically biocompatible and biodegradable, and their physical properties can be tuned via the peptide sequence design [43]. Therapeutic agents, including small-molecule drugs, proteins, or even therapeutic nanoparticles, can be physically encapsulated within the hydrogel matrix during its formation or loaded post-fabrication [44]. A key advantage of peptide hydrogels in oncology is their potential for injectable, localized delivery directly into the tumor site or the resection cavity after surgery. This can provide sustained high local concentrations of the therapeutic agent, minimizing systemic exposure and associated toxicity [44]. Furthermore, peptide hydrogels can be designed to be stimuli-responsive, releasing their payload in response to specific cues within the tumor microenvironment, such as pH changes, enzyme activity, or externally applied triggers like light or temperature [44]. Recent research has explored peptide hydrogels for the sustained release of chemotherapeutics, immunomodulatory agents, or as scaffolds for delivering engineered cells in cancer treatment [44,45].

While non-bioactive peptide systems provide significant advantages in drug delivery through their structural attributes, the incorporation of peptides with inherent biological functions offers a further leap in therapeutic sophistication. The subsequent section will focus on these bioactive peptide-based nanoparticles, where peptides actively contribute to targeting, cellular uptake, or therapeutic effect.

## 4. Bioactive Peptide-Based Nanoparticles for Tumor Therapy

### 4.1. Introduction

Contrasting with systems where peptides primarily provide structure, bioactive peptide-based nanoparticles incorporate peptide sequences that actively participate in the therapeutic strategy [46]. These peptides are not merely passive components but are designed to perform specific biological functions, such as recognizing and binding to targets on cancer cells or within the TME (targeting peptides), facilitating the entry of the nanoparticle and its cargo into cells (penetrating peptides), or directly exerting an anticancer effect (therapeutic peptides). This approach represents a move towards greater precision and efficacy, aiming to overcome the limitations of non-specific therapies and passive nanoparticle accumulation. By integrating specific biological functions via the peptide component, these nanoparticles can achieve enhanced tumor localization, improved cellular uptake, direct cell killing, or modulation of the tumor immune landscape. Often, these functionalities are combined, with peptides exhibiting dual roles (e.g., targeting and penetration) or multiple bioactive peptides being incorporated into a single nanosystem, reflecting a trend towards increasingly sophisticated and multifunctional designs [47]. This section explores the major classes of bioactive peptides utilized in nanoparticle systems for cancer therapy.

### 4.2. Targeting Peptides

**Figure 4:**
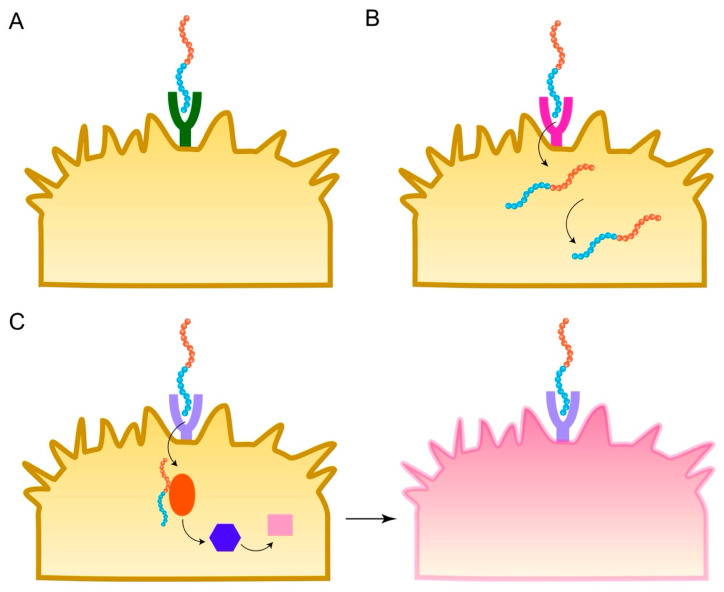
Mechanisms of action for bioactive peptide nanoparticles. (**A**) Targeting peptides bind to receptors. (**B**) CPPs facilitate entry across the cell membrane. (**C**) Therapeutic peptides trigger tumor cell death.

Active targeting is a key strategy to enhance the specificity and efficacy of nanoparticle-based cancer therapies, aiming to overcome the limitations and heterogeneity of the passive EPR effect [9]. Targeting peptides, also known as tumor-homing peptides, are short amino acid sequences displayed on the nanoparticle surface that are designed to bind with high affinity and specificity to molecular targets—typically receptors or other proteins—that are overexpressed on the surface of cancer cells or associated stromal and vascular cells within the TME, while being less abundant on normal tissues [4] (Figure 4A). Table 1 summarizes the targeting peptides, their receptors, and targeted cancers. 

The binding of the targeting peptide to its cognate receptor facilitates the selective accumulation of the nanoparticle at the tumor site and often triggers receptor-mediated endocytosis, leading to efficient internalization of the nanoparticle and its therapeutic payload into the target cells [48]. This targeted delivery mechanism aims to increase the local concentration of the therapeutic agent within the tumor, thereby enhancing its anticancer effect while minimizing exposure and toxicity to healthy organs [7].

#### 4.2.1. Discovery of Targeting Peptides

The identification of suitable targets and corresponding peptide ligands is crucial. Phage display technology has been instrumental in discovering novel tumor-homing peptides by screening vast peptide libraries (displaying peptides on the surface of bacteriophages) against cancer cells, tissues, or specific receptors [49]. This technique allows for the rapid selection of peptides with high binding affinity and specificity from billions of candidates [50]. Other methods, such as “one-bead one-compound” (OBOC) libraries (where each bead in a large library displays a unique chemical compound), as well as computational approaches, are also employed [51].

#### 4.2.2. Structural Considerations: Linear vs. Cyclic Peptides

Targeting peptides can be linear or cyclic. While linear peptides are often simpler to synthesize, cyclic peptides generally offer significant advantages for nanoparticle functionalization [13]. Cyclization restricts the peptide’s conformational flexibility, which can lead to the following [13]:

Enhanced Binding Affinity and Selectivity: By pre-organizing the peptide into a conformation that fits the target receptor better, cyclization often increases binding affinity (nM to µM range for peptides vs. pM to nM for antibodies) and specificity compared to the linear counterpart [13].

Improved Stability: The cyclic structure protects the peptide backbone from degradation by exopeptidases and can increase resistance to endopeptidases, leading to a longer half-life in vivo [13].

Better-Defined Structure: The constrained conformation facilitates structural analysis and rational design [52]. Cyclization can be achieved through various chemical strategies, including disulfide bonds (e.g., using cysteine residues) or amide linkages [13]. For example, cyclic RGD peptides (cRGD) show superior binding efficacy to integrins compared to linear RGD [53].

#### 4.2.3. Prominent Examples of Targeting Peptides for Nanoparticle Systems

Numerous targeting peptides have been identified and utilized in conjunction with nanoparticles for cancer therapy. Some prominent examples include the following:

##### RGD Peptides

The Arginine–Glycine–Aspartic acid (RGD) sequence is perhaps the most widely studied targeting motif. It binds primarily to integrins, particularly αvβ3 and αvβ5 subtypes, which are transmembrane receptors involved in cell adhesion, migration, and signaling [4]. These integrins are frequently overexpressed on the surface of various cancer cells (e.g., melanoma, glioblastoma, breast, prostate, ovarian cancer) and on endothelial cells of the tumor neovasculature, playing key roles in tumor growth, angiogenesis, and metastasis [13]. RGD peptides, especially cyclic versions (cRGD) which exhibit enhanced stability against proteolysis and often higher binding affinity and selectivity due to conformational constraints, are conjugated to nanoparticles carrying chemotherapeutics (e.g., paclitaxel, doxorubicin, cisplatin), gene therapies, or imaging agents to target these integrins [2]. The incorporation of RGD peptides can also facilitate the self-assembly of nanodrugs and influence drug release kinetics [53].

##### NGR Peptides

The Asn-Gly-Arg (NGR) motif targets aminopeptidase N (APN/CD13), a cell surface metalloprotease that is overexpressed on angiogenic blood vessels and certain tumor cells [54]. NGR-functionalized nanoparticles have been developed to deliver cytotoxic drugs (e.g., doxorubicin, docetaxel) or other therapeutic agents specifically to the tumor vasculature or CD13-positive tumor cells [55]. Some NGR peptides possess a C-end Rule (CendR) motif, a C-terminal sequence motif that, when exposed, can mediate tissue penetration by binding to neuropilin-1, which, after initial binding and potential cleavage, can interact with neuropilin-1 (NRP-1) to enhance tumor penetration [55]. Dual-targeting strategies combining NGR with other peptides (e.g., BBB-penetrating peptides) have been explored for treating brain tumors like glioma [56]. Non-covalent strategies for attaching NGR peptides to nanoparticles have also been developed [55].

##### LyP-1 Peptides

The cyclic peptide LyP-1 (CGNKRTRGC) targets the protein p32 (also known as gC1qR or HABP1), which is upregulated on the surface of various tumor cells (e.g., breast cancer, osteosarcoma), tumor-associated macrophages, and tumor lymphatic vessels, particularly under conditions of cellular stress such as hypoxia or heat treatment [57]. LyP-1 not only homes to tumors but also possesses cell-penetrating properties, facilitating intracellular delivery [57]. It has been used to functionalize nanoparticles carrying chemotherapeutics (e.g., doxorubicin) [58], imaging agents [59], or even oncolytic adenoviruses expressing therapeutic proteins (e.g., sTGFβRIIFc) [57]. Cooperative nanosystems involving LyP-1-targeted liposomes and heat-generating gold nanorods have also been reported [60]. LyP-1-functionalized lipid–polymer composite nanoparticles showed enhanced tumor accumulation in osteosarcoma models [59].

##### CREKA Peptides

The linear peptide CREKA (Cys-Arg-Glu-Lys-Ala) specifically binds to fibrin and fibrin–fibronectin complexes, which are abundant in the tumor stroma, associated with tumor vasculature (microthrombi), and play a role in metastasis [61]. By targeting these TME components rather than cancer cells directly, CREKA-functionalized nanoparticles (e.g., liposomes, PEG hydrogels) can accumulate and be retained within the tumor mass [62]. This strategy has been employed to deliver drugs (e.g., doxorubicin, ticagrelor) or imaging agents for treating solid tumors like glioblastoma and potentially inhibiting metastasis by targeting tumor-associated clots [62]. CREKA-conjugated PEG hydrogel nanoparticles showed enhanced cellular uptake and fibrin binding ability [63]. Liposomal nanoparticles functionalized with CREKA have been used to deliver platelet inhibitors (ticagrelor) to tumor microthrombi to potentially inhibit metastasis [64].

##### Other Targeting Peptides

A variety of other peptide ligands are used, targeting receptors like HER2 using peptides such as HER2pep (YCDGFYACYMDV) or THP (WNLPWYYSVSPTC) for HER2-positive breast cancer [19]; the Transferrin Receptor 1 (TfR1), which is widely overexpressed in proliferating cancer cells, using specific peptides or by employing the natural TfR1 ligand, ferritin, as the nanoparticle itself [65]; receptors for hormones or neuropeptides like bombesin, neuropeptide Y (NPY), or somatostatin (using analogs like octreotide) which are overexpressed in certain neuroendocrine and other tumors [2]; and targets within the tumor-associated microbiome or on cancer-associated fibroblasts.

The selection of the appropriate targeting peptide depends heavily on the specific cancer type and the expression profile of targetable markers. The development of multivalent or multi-ligand nanoparticles, displaying multiple copies of a single peptide or combinations of different peptides, is also being explored to enhance binding avidity and specificity, or target multiple pathways simultaneously [46].

### 4.3. Penetrating Peptides (CPPs)

While targeting peptides enhance accumulation at the tumor site and binding to cancer cells, delivering the therapeutic payload inside the cells remains a critical barrier. Cell membranes are selectively permeable, restricting the passage of many molecules, especially large ones like nanoparticles or hydrophilic drugs [66]. Cell-penetrating peptides (CPPs) are a class of short peptides, typically 5–30 amino acids long (though sometimes up to 40) and often possessing a net positive charge or amphipathic character, that have the remarkable ability to traverse biological membranes and facilitate the intracellular delivery of various molecular cargoes covalently or non-covalently associated with them [15] (Figure 4B). They are efficient tools for delivering small molecules, DNA, siRNA, proteins, and nanoparticles into cells and tissues [67].

#### 4.3.1. Mechanisms of Cellular Uptake

The mechanisms by which CPPs mediate cellular uptake are diverse and not fully elucidated, often depending on the specific CPP sequence, the nature and size of the cargo, the cell type, and concentration [67]. Two main pathways are generally described as follows.

##### Direct Penetration (Translocation)

Some CPPs are thought to directly cross the lipid bilayer of the cell membrane in an energy-independent manner. This may involve transient pore formation, membrane destabilization, or interactions with membrane lipids [67]. Cationic CPPs interact electrostatically with negatively charged components of the cell surface (like proteoglycans and phospholipids), while hydrophobic or amphipathic CPPs interact with the lipid bilayer [67]. Direct translocation may be favored at higher CPP concentrations [47].

##### Endocytosis

Many CPPs and their cargoes are internalized via various energy-dependent endocytic pathways, including macropinocytosis, clathrin-mediated endocytosis, and caveolae-mediated endocytosis [12]. This pathway is often favored at lower CPP concentrations [47]. Following internalization, the CPP–cargo complex is enclosed within endosomes. A significant challenge then becomes escaping the endosome to release the cargo into the cytoplasm or target specific organelles, as failure to do so can lead to lysosomal degradation [47]. Some CPPs possess endosomolytic properties that facilitate this escape.

**Table 1:** Examples of targeting peptides, their receptors, and targeted cancers.

Peptide Name (Sequence Example)	Target Receptor/Molecule	Receptor Location	Delivery Vehicle Type(s) Used	Delivery Mechanism/Key Outcome	Example Cancer Types	Ref.
RGD (e.g., cyclic RGDfK)	Integrins (αvβ3, αvβ5)	Tumor cells, tumor vasculature	Lipid–polymer hybrid nanoparticles, ferritin (fusion), Inulin multimethacrylate NPs	Receptor-mediated endocytosis/enhanced drug accumulation, delivery	Glioblastoma, melanoma, breast, prostate, ovarian, lung	[2,54,65]
NGR (Asn-Gly-Arg)	Aminopeptidase N (CD13)	Tumor vasculature, some tumor cells	ND	Tumor vasculature-targeting/drug delivery	Glioma, breast, fibrosarcoma	[54]
LyP-1 (CGNKRTRGC)	p32 (gC1qR/HABP1)	Tumor cells, macrophages, lymphatics	Oncolytic adenovirus (genetically modified fiber)	Receptor binding, enhanced viral tropism and replication in cancer cells/antitumor response, inhibited metastasis, augmented ICI therapy	Breast, osteosarcoma, prostate	[57]
CREKA (Cys-Arg-Glu-Lys-Ala)	Fibrin/Fibrin–Fibronectin	Tumor stroma, tumor microthrombi	Amino dextran-coated iron oxide (SPIO) nanoparticles	ND	Glioblastoma, general solid tumors (anti-metastasis)	[61]
HER2pep (YCDGFYACYMDV)	HER2	Tumor cells	Liposomes (with oligolysine/EG linkers), Nanoparticles	Receptor-mediated endocytosis/enhanced cellular uptake, targeted drug delivery (e.g., doxorubicin, capecitabine)	HER2+ breast cancer	[68]
THP (WNLPWYYSVSPTC)	HER2	Tumor cells	Liposomes	Receptor binding/enhanced drug uptake, interference with apoptotic signaling	HER2+ breast cancer	[68]
Ferritin protein	Transferrin receptor 1 (TfR1/CD71)	Tumor cells, BBB	Ferritin nanocage	TfR1-mediated transcytosis/endocytosis; enhanced tumor/brain accumulation of drugs (DOX, paclitaxel, cisplatin, etc.)	Various proliferating cancers, brain tumors	[65]
Octreotide	Somatostatin receptors	Tumor cells	Nanoparticles (self-assembled with drug)	Receptor-mediated targeting/drug delivery (e.g., doxorubicin)	Neuroendocrine tumors	[2]
P-LPK (LPKTVSSDMSLN)	Unspecified CRC receptor	Tumor cells	Self-assembled peptide–drug nanoparticles (LPK-PTX NPs)	Targeted delivery, enhanced intracellular internalization and tumor accumulation/improved tumor cytotoxicity, enhanced antitumor activity in vivo, decreased systemic toxicity.	Colorectal cancer (CRC)	[8]
C-peptide (endostatin-derived)	Integrin αvβ3	Tumor cells	Solid lipid nanoparticles (SLNs)	Integrin targeting; enhanced cytotoxicity, inhibited cell migration, pH-dependent drug release/tumor volume reduction, prevented metastasis, apoptosis induction.	Triple-negative breast cancer (TNBC)	[4]

ND: Not described in the cited reference.

In the context of nanoparticle-based cancer therapy, CPPs are primarily used as molecular transporters to enhance the cellular uptake of nanoparticles or nanoparticle–drug complexes [15]. By conjugating CPPs to the nanoparticle surface (covalently) or forming non-covalent complexes (e.g., through electrostatic interactions between cationic CPPs and negatively charged cargo like nucleic acids or anionic NPs), the efficiency of internalization into cancer cells can be significantly increased [67]. This strategy is particularly valuable for delivering membrane-impermeable drugs, proteins, or gene therapeutics (siRNA, DNA) 1. Examples of CPPs used in cancer nanomedicine include the well-known Tat peptide derived from the HIV-1 Tat protein [61], Antennapedia (Antp) homeodomain peptide [67], polyarginine sequences, pVEC [47], and others like Z12 [47], CPP44/CPP33 1, and even peptides with dual targeting/penetrating roles like LyP-1 [57]. Recent work has also explored CPP-like peptides conjugated to photosensitizers (Chlorin e6) and targeting ligands (anti-PD-L1 peptide) that self-assemble into nanoparticles with enhanced permeability [69].

#### 4.3.2. Strategies to Enhance Specificity and Overcome Limitations

Despite their promise, CPPs face significant challenges for systemic in vivo application.

Lack of Specificity: A major limitation is their lack of cell specificity; they tend to enter most cell types, which can lead to off-target accumulation and toxicity if delivering potent therapeutics [1]. Strategies to address this include designing tumor-activated CPPs (e.g., cleaved by tumor-specific enzymes) or combining CPPs with tumor-targeting ligands on the same nanoparticle to achieve synergistic targeting and uptake [1].

Poor Stability and Pharmacokinetics: CPPs are susceptible to protease degradation in serum and can be rapidly cleared, leading to short half-lives [1]. Chemical modifications such as cyclization, the incorporation of unnatural amino acids, lipidation, or PEGylation can improve stability and pharmacokinetic properties [1]. Conjugation to nanoparticles or carriers can also offer protection [67].

Immunogenicity: CPPs can potentially elicit immune responses [1]. Modifications like PEGylation can help reduce immunogenicity [47].

Endosomal Entrapment: Cargo delivered via endocytosis can become trapped in endosomes and degraded in lysosomes, preventing it from reaching its intracellular target [1]. Designing CPPs with inherent endosomal escape capabilities or co-delivering endosomolytic agents are strategies to overcome this [1].

Tissue Penetration: While CPPs enhance cellular uptake, penetration into dense solid tumor tissues can still be limited [47].

Ongoing research focuses on rational design and chemical modifications to develop next-generation CPPs with improved stability, selectivity, tissue penetration, and endosomal escape capabilities for safer and more effective cancer therapy [1].

### 4.4. Therapeutic Peptides

Beyond serving as targeting or delivery aids, peptides themselves can possess intrinsic anticancer activity [17] (Figure 4C). These therapeutic peptides, often termed anticancer peptides (ACPs), exert their effects through diverse mechanisms, including inducing apoptosis, disrupting cell membranes, inhibiting angiogenesis, modulating critical signaling pathways, or stimulating an antitumor immune response [18]. Incorporating these bioactive peptides into nanoparticle formulations can overcome their inherent limitations (like poor stability and a short half-life) and enhance their delivery to tumor sites, thereby amplifying their therapeutic potential [4]. Two major classes of therapeutic peptides relevant to nanoparticle delivery are pro-apoptotic peptides and immunomodulatory peptides. Table 2 summarizes the therapeutic peptides and their mechanisms of action.

#### 4.4.1. Pro-Apoptotic Peptides

Inducing programmed cell death, or apoptosis, is a highly desirable mechanism for cancer therapy as it leads to the controlled elimination of malignant cells with minimal inflammation and damage to surrounding tissues [70]. Pro-apoptotic peptides are designed to specifically trigger or enhance apoptotic pathways within cancer cells [70]. Cancer cells often develop resistance to apoptosis by upregulating anti-apoptotic proteins (e.g., Bcl-2, Mcl-1) or acquiring mutations in key regulators like p53. Pro-apoptotic peptides aim to overcome this resistance by directly targeting critical components of the apoptotic machinery.

##### Mechanisms of Apoptosis Induction

Common strategies by which pro-apoptotic peptides induce cell death include the following [1]:

Mitochondrial Disruption: Mitochondria play a central role in the intrinsic apoptosis pathway. Peptides can be designed to target and permeabilize mitochondrial membranes, leading to the release of pro-apoptotic factors like cytochrome c. A well-known example is the cationic, amphipathic peptide D[KLAKLAK]_2_, which selectively disrupts mitochondrial membranes [71]. To deliver this peptide specifically to tumors, it has been conjugated to tumor-homing peptides (like CGKRK, which itself shows mitochondrial localization) and incorporated into nanoparticles (e.g., iron oxide NPs for imaging capabilities). The systemic administration of these targeted nanoparticles showed significant tumor regression in preclinical glioblastoma models [71]. Other cytotoxic peptides, like (KLAKLAK)_2_, have also been conjugated to polymers to form self-assembling nanoparticles [2]. Melittin, a cytolytic peptide from bee venom, also induces apoptosis and cell cycle arrest, partly through mitochondrial pathways, and its delivery via nanoparticles is being explored [16].

Modulating Bcl-2 Family Proteins: The balance between pro-apoptotic (e.g., Bax, Bak, Bim) and anti-apoptotic (e.g., Bcl-2, Bcl-xL, Mcl-1) proteins of the Bcl-2 family is critical for controlling the intrinsic pathway. Stapled peptides, which are conformationally constrained by a synthetic brace, have been developed to mimic the BH3 domains of pro-apoptotic proteins [70]. These peptides can bind to and inhibit anti-apoptotic proteins like Mcl-1 (e.g., SAHB_D) or Bcl-2/Bcl-xL (e.g., BIM-SAHB_A), thereby unleashing apoptosis [47]. Nanoparticle delivery could potentially enhance the intracellular delivery and stability of these stapled peptides.

Reactivating the p53 Pathway: The tumor suppressor protein p53 is a master regulator of apoptosis, but it is often mutated or inhibited in cancer. Peptides can be used to restore p53 function. The peptide p28, derived from the bacterial protein azurin, stabilizes p53 and induces cell cycle arrest and apoptosis in various cancer types, showing promise in early clinical trials [70]. Stapled peptides like ATSP-7041 and ALRN-6924 target the p53 inhibitors MDM2 and MDMX, releasing p53 to trigger apoptosis; ALRN-6924 has also entered clinical investigation [70].

Interfering with DNA Synthesis/Repair: Some peptides can directly interfere with DNA replication or repair mechanisms, leading to DNA damage accumulation and subsequent apoptosis [70]. For example, peptides targeting Holliday junctions or the C-terminal domain of BRCA1 have been reported [70]. Peptides derived from insect hemolymph have shown the targeted inhibition of DNA synthesis in cancer cells [72].

Nanoparticle delivery platforms are crucial for translating the potential of these pro-apoptotic peptides into effective therapies by improving their stability, solubility, tumor targeting, and intracellular delivery.

#### 4.4.2. Immunomodulatory Peptides

Cancer immunotherapy aims to harness the patient’s own immune system to fight cancer [17]. However, tumors often evolve mechanisms to evade immune surveillance and create an immunosuppressive microenvironment [17]. Immunomodulatory peptides are designed to counteract these mechanisms, boost antitumor immune responses, or serve as specific targets for the immune system [17]. Given that current immunotherapies like checkpoint inhibitors only benefit a subset of patients (~30%) [17], peptide-based strategies offer avenues to improve response rates and overcome resistance. Nanoparticles play a vital role in delivering these immunomodulatory peptides effectively, protecting them from degradation, targeting them to immune cells or the TME, and potentially acting as adjuvants themselves [6].

##### Peptide Vaccines

These vaccines use synthetic peptides corresponding to tumor-associated antigens (TAAs) or tumor-specific antigens (TSAs), including neoantigens derived from tumor mutations, to elicit targeted immune responses [15]. The goal is to prime and activate cytotoxic T lymphocytes (CTLs, CD8^+^ T cells) that can recognize and kill cancer cells displaying these antigens on their MHC class I molecules, often with the help of CD4^+^ T helper cells activated by peptide presentation on MHC class II 16. Both short peptides (8–12 amino acids, primarily for MHC-I) and synthetic long peptides (SLPs, ≥20 amino acids, processed for both MHC-I and MHC-II) are used [73]. Short peptides tend to have short half-lives and may induce tolerance if not presented properly by APCs, while SLPs are generally more stable and immunogenic as they require processing by APCs, leading to both CD4+ and CD8+ T cell activation [73]. Another strategy to enhance vaccine potency involves the design of tandem peptide vaccines or multi-epitope long peptides (MELPs). These constructs typically link multiple distinct peptide epitopes—often encompassing different tumor-associated antigens (TAAs), CD4+ helper epitopes, and CD8+ cytotoxic T lymphocyte (CTL) epitopes—into a single polypeptide chain, sometimes separated by specific cleavable linkers or spacers [74,75]. The rationale is that a single construct can simultaneously present diverse antigens or combine crucial T-helper and CTL epitopes, thereby eliciting a broader and potentially more robust and durable anti-tumor immune response compared to single epitopes [74,75]. These tandem peptides can be delivered using various systems, including nanoparticle formulations, to protect them from degradation and enhance their uptake and processing by APCs [76]. Peptide vaccines generally have a good safety profile but often suffer from low immunogenicity [73]. Nanoparticle delivery systems (including self-assembling peptide structures) are crucial for enhancing immunogenicity by protecting peptides, facilitating uptake by antigen-presenting cells (APCs) like dendritic cells (DCs), co-delivering adjuvants (e.g., TLR agonists), and promoting sustained antigen presentation [1]. Personalized peptide vaccines based on patient-specific neoantigens represent a promising frontier [73].

##### Immune Checkpoint Blockade Peptides

**Figure 5:**
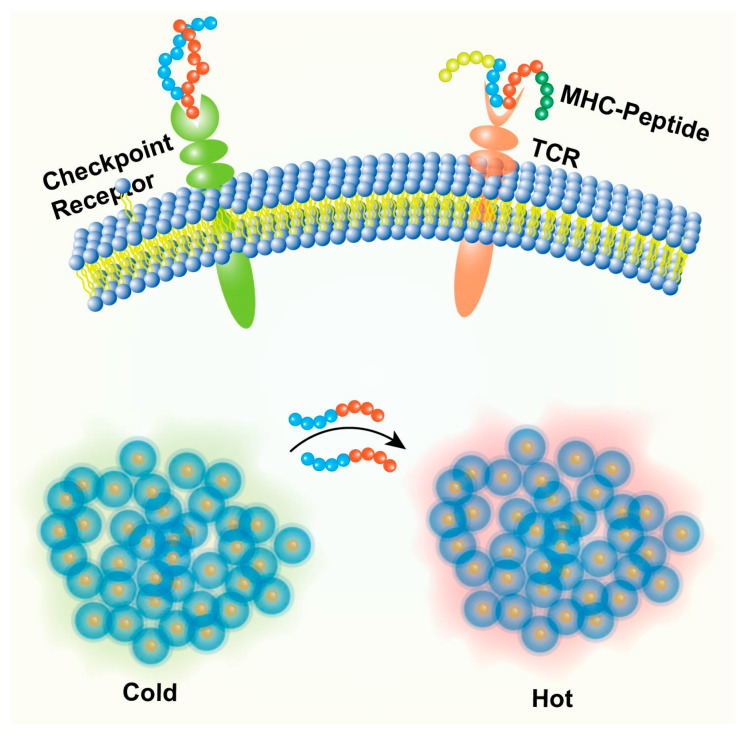
Peptide nanoparticles in cancer immunotherapy. Nanoparticle delivering peptide antigens +/− adjuvants to an antigen-presenting cell (APC) leading to T cell activation. Nanoparticle delivering peptides that block PD-1/PD-L1 or other checkpoint interactions between T cells and tumor cells. Nanoparticles targeting and modulating immunosuppressive cells (e.g., M2 TAMs) or delivering cytokines within the TME.

Immune checkpoints normally regulate immune responses but are often exploited by tumors to suppress antitumor immunity [17]. While monoclonal antibodies targeting these checkpoints are clinically successful, peptides offer potential advantages like better tumor penetration and lower cost [77]. Peptides have been developed to block the PD-1/PD-L1 interaction, thereby restoring T cell and NK cell activity against the tumor [18] (Figure 5). Nanoparticles functionalized with or encapsulating these peptides can improve their delivery and efficacy [18]. Peptides targeting other checkpoints like TIGIT (e.g., DTBP-3) are also under investigation [18].

##### Modulation of Innate and Adaptive Immune Cells

Peptides can directly activate or modulate innate immune cells crucial for antitumor responses (Figure 5). This includes peptides that activate DCs, enhance NK cell cytotoxicity, or repolarize tumor-associated macrophages (TAMs) from a pro-tumor M2 phenotype to an anti-tumor M1 phenotype (e.g., using M2pep to deliver therapeutics to M2 TAMs) [15]. Peptides activating the cGAS-STING pathway can induce type I interferon production, bridging innate and adaptive immunity [18]. Nanoparticle delivery can target these peptides specifically to the relevant immune cell populations within the TME. Peptides can also be designed to activate T cells or stimulate B cells to produce anti-tumor antibodies [18]. Some peptides, like LfcinB, can regulate immune cells (CD4+, CD8+, NK cells) and increase cytokine production [72].

##### Targeting Immunosuppressive Mechanisms

Peptides can be designed to disrupt immunosuppressive interactions within the TME (Figure 5). For example, peptides designed to block the CD47-mediated ‘don’t eat me’ signal through CD47 targeting or its receptor SIRPα on macrophages can enhance phagocytosis of cancer cells [18]. Nanoparticle formulation can increase the local concentration and effectiveness of such peptides.

##### Self-Assembling Immunomodulators

Peptides can be designed to self-assemble into nanostructures (nanofibers, hydrogels) that inherently possess adjuvant properties or can serve as depots for the sustained release of antigens, adjuvants, or other immunomodulatory molecules within the TME or lymph nodes, thereby shaping the local immune response [18]. These self-assembling systems can enhance uptake by APCs and promote stronger immune activation [18].

The integration of immunomodulatory peptides with nanoparticle platforms offers a powerful and versatile approach to enhance cancer immunotherapy, potentially overcoming resistance, improving response rates, and enabling combination strategies.

The diverse strategies and specific examples discussed highlight the immense versatility and therapeutic promise of bioactive peptide-based nanoparticles in oncology. To synthesize these findings and consider the path forward, the concluding section will address the current status, ongoing challenges, and future perspectives of this dynamic field.

## 5. Conclusions and Discussion

### 5.1. Synthesis of Current Status

The field of peptide-based nanoparticles has emerged as a highly promising and rapidly advancing frontier in cancer therapy [1]. By integrating the inherent advantages of peptides—such as biocompatibility, specificity, and synthetic versatility—with the drug-delivery capabilities of nanotechnology, these systems offer “novel strategies to address the shortcomings of conventional treatments and earlier nanomedicine approaches. Research has demonstrated the remarkable potential of peptide nanoparticles across a spectrum of applications, from improving the pharmacokinetics and reducing the toxicity of established chemotherapeutics using non-bioactive polypeptide, dendrimer, or ELP carriers [32], to enabling highly precise therapeutic interventions through bioactive peptides.

Significant progress has been made in designing peptides that actively target specific receptors overexpressed on cancer cells or within the TME, enhancing drug accumulation and cellular uptake [46]. Cell-penetrating peptides have proven effective in overcoming cellular barriers to deliver diverse cargoes intracellularly [67]. Furthermore, the development of therapeutic peptides delivered via nanoparticles, capable of directly inducing apoptosis or modulating the immune system, represents a major stride towards targeted and potentially curative treatments [18]. The ability to engineer peptide self-assembly through a deep understanding of driving forces like amphiphilicity, non-covalent interactions, and metal coordination allows for the creation of diverse nanostructures with tailored properties [2]. Preclinical studies across numerous cancer models have validated the potential of these systems, showcasing enhanced efficacy, reduced toxicity, and the ability to address challenges like drug resistance [2].

### 5.2. Major Challenges and Hurdles

Despite the considerable enthusiasm and promising preclinical results, the translation of peptide-based nanoparticles into routine clinical practice remains a significant challenge, with only a limited number of systems progressing successfully through clinical trials [78]. Several critical hurdles must be addressed. Table 3 outlines the major challenges and corresponding strategies for peptide nanoparticle applications in cancer therapy. 

In Vivo Stability and Pharmacokinetics: Peptides are inherently susceptible to rapid degradation by proteases in biological fluids, and both peptides and nanoparticles can be quickly cleared from circulation by the kidneys or the reticuloendothelial system (RES), resulting in short half-lives and limiting the time available for tumor accumulation [4]. While nanoparticle encapsulation offers protection, ensuring the long-term stability of the entire construct and preventing premature drug leakage in vivo remains complex [19].

Delivery Efficiency and Tumor Penetration: Achieving sufficient accumulation within the tumor mass is often hindered by the heterogeneity of the EPR effect and the complex biological barriers presented by the TME [9]. High interstitial fluid pressure, dense extracellular matrix, and abnormal vasculature can severely limit the penetration of nanoparticles, especially larger ones, beyond the perivascular regions, leaving deeper tumor cells untreated [12]. Crossing specialized barriers like the blood–brain barrier (BBB) to treat brain tumors poses an additional, formidable challenge [46]. Furthermore, even after reaching the target cell, efficient endosomal escape is required for intracellularly active payloads [47].

**Table 2:** Examples of therapeutic peptides and their mechanisms of action.

Peptide Class	Specific Peptide Example (Name/Sequence)	Mechanism of Action	Target Pathway/ Molecule	Nanoparticle/Delivery Vehicle Type	Relevant Tumor Type(s) Example	Ref.
Pro-apoptotic	D[KLAKLAK]_2_	Mitochondrial membrane disruption	Mitochondria	Iron oxide nanoparticles (“nanoworms”)	Glioblastoma (GBM)	[71]
	p28 (azurin fragment 50–77)	p53 stabilization, cell cycle arrest, apoptosis induction	p53 pathway	Used as CPP/single agent in trials	Solid tumors (GBM, CNS, hepatocellular carcinoma in trials), breast cancer (preclinical)	[70]
	ALRN-6924 (stapled peptide)	Inhibition of p53 suppressors, p53 reactivation	MDM2, MDMX	Peptide therapeutic (stapled)	Solid tumors, lymphomas, AML, ER+ breast cancer	[47,70]
	BIM-SAHB_A (stapled peptide)	Inhibition of anti-apoptotic proteins	Bcl-2, Bcl-xL, Mcl-1	Stapled peptide	Hematologic cancers	[47]
	Melittin	Membrane lysis, apoptosis induction, cell cycle arrest	Cell membrane, mitochondria, CDK pathways	Lipodisks, lipid nanoparticle, polymer conjugates (PEG), graphene complexes, fusion proteins, polymeric NPs (MpG@LPN), bioinspired lipoproteins, micelles	Breast, bladder, gastric, colorectal, liver, lung, melanoma, glioma	[16]
Immunomodulatory (vaccine)	TAA/TSA/neoantigen peptides (various)	Antigen presentation, T cell/B cell activation	MHC Class I/II, T cell receptors, B cell receptors	Peptide + adjuvant, self-healing microcapsules, phage display vectors	Melanoma, TNBC, glioma, CRC, NSCLC, pancreatic, ovarian, HER2+ breast cancer	[18]
Immunomodulatory (checkpoint)	Anti-PD-L1 peptides (various)	Blockade of inhibitory PD-1/PD-L1 interaction	PD-1/PD-L1 axis	Self-assembling peptides (TAP), oral microemulsion (OPBP-1), multi-stimulus NPs (MAPN), PD-L1 peptide (PD-NPs).	various solid tumors (preclinical models, including PDX and anti-PD-1 resistant models)	[18]
	DTBP-3 (anti-TIGIT peptide)	Blockade of inhibitory TIGIT/PVR interaction	TIGIT/PVR axis	D-peptide	Anti-PD-1 resistant tumor models	[18]
Immunomodulatory (cell modulator)	M2pep	Targeting M2 macrophages for payload delivery	Scavenger receptor B type 1 (SR-B1) on M2 TAMs	Cyclodextrin-siRNA NPs, phage display strategy, ZrMOF nanoparticles	Prostate cancer, melanoma	[18,79,80]
	Pep-20 (anti-CD47 peptide)	Blockade of “don’t eat me” signal, enhances phagocytosis	CD47/SIRPα axis	ZrMOF nanoparticles, phage display strategy	Ovarian, breast, colon cancer, glioblastoma and acute lymphoblastic leukemia	[80,81]
	(STING agonist peptides)	Activation of innate immunity, type I IFN production	cGAS-STING pathway	Self-assembling peptide hydrogels (STINGel); multi-stimulus NPs (MAPN). MnO_2_–melittin–manganese NPs (melittin induces STING), ZrMOF nanoparticles	Various solid tumors (preclinical)	[18,80]

Specificity and Off-Target Effects: While active targeting aims to improve specificity, challenges remain. Target receptors may also be expressed at lower levels on healthy tissues, resulting in potential off-target binding and side effects [9]. Tumor heterogeneity means that not all cancer cells within a tumor may express the target receptor at sufficient levels, limiting therapeutic efficacy [9]. Moreover, the non-specific uptake of nanoparticles by the RES can reduce the dose available for the tumor and potentially cause organ toxicity [7]. CPPs, in particular, often lack inherent tissue specificity [67].

Immunogenicity: Although generally considered less immunogenic than antibodies, both peptides and nanoparticle carriers can potentially elicit immune responses, leading to the formation of antibodies that neutralize the therapeutic agent or cause adverse reactions [17]. Balancing stability and efficacy with minimal immunogenicity is a critical design consideration.

Complexity, Scalability, and Cost: Designing and synthesizing multifunctional peptide nanoparticles often involves complex chemistry and multiple components. Ensuring batch-to-batch reproducibility and developing scalable, cost-effective manufacturing processes compliant with Good Manufacturing Practice (GMP) standards are significant hurdles for industrial development and clinical translation [34].

Clinical Translation Gap: The discrepancy between the vast number of promising preclinical studies and the paucity of clinically approved peptide nanoparticle therapies highlights the difficulties in translating laboratory findings into tangible patient benefits [78]. This gap stems from the complexity of in vivo biological interactions, the limitations of current animal models to predict human responses, and the stringent regulatory requirements for nanomedicines.

#### Regulatory and Manufacturing Hurdles

Beyond the biological and chemical challenges, significant practical hurdles exist in bringing peptide nanoparticles to the clinic. Manufacturing these complex systems on a large scale while maintaining consistent quality and batch-to-batch reproducibility is difficult and often expensive [12]. The synthesis of peptides, especially modified or long sequences, and the subsequent nanoparticle formulation require specialized processes that adhere to strict Good Manufacturing Practice (GMP) guidelines [12]. Furthermore, the regulatory pathway for nanomedicines is still evolving and can be complex, requiring extensive characterization and safety data to satisfy agencies like the FDA [12]. The inherent complexity of multifunctional nanoparticles adds layers to the characterization and regulatory approval process [17]. Overcoming these manufacturing and regulatory challenges is essential for successful clinical translation.

Addressing these multifaceted challenges requires continued innovation in peptide engineering, nanoparticle design, and TME manipulation strategies, and the development of more predictive preclinical models.

**Table 3:** Summary of challenges and potential solutions for peptide nanoparticles in cancer therapy.

Challenge Category	Specific Challenge Description	Potential Solutions/Strategies	Ref.
Stability/PK	Peptide degradation (proteolysis), rapid clearance, short half-life	Peptide modification (cyclization, unnatural AAs, stapling), PEGylation, encapsulation/protection, High MW ELPs, fusion to carrier proteins	[3]
	NP instability, premature drug leakage	Cross-linking NP core/shell, optimizing formulation stability (e.g., using cholesterol in liposomes), controlled release mechanisms	[34]
Delivery/penetration	Low tumor accumulation (EPR limits), poor penetration into tumor	Active targeting ligands (peptides), tumor-penetrating peptides (e.g., iRGD, CPPs), TME normalization/modulation strategies, smaller NP size, stimuli-responsive penetration enhancement	[46]
	Crossing biological barriers (e.g., BBB)	Specific transporter-targeting peptides (e.g., TfR), rceptor-mediated transcytosis strategies, biomimetic approaches (e.g., RBC coating)	[46]
	Endosomal escape for intracellular cargo	Incorporating endosomolytic peptides/agents, pH-responsive NPs, photochemical internalization	[47]
Specificity/toxicity	Off-target binding/uptake, toxicity to healthy tissues	High-affinity/specificity targeting peptides, targeting TME components, prodrug strategies, stimuli-responsive activation/release in tumor, PEGylation/stealth coatings to reduce RES uptake	[7]
	Tumor heterogeneity (receptor expression)	Multi-targeting strategies, targeting common markers, theranostic approaches for patient selection, adaptive therapies	[46]
Immunogenicity	Immune response to peptide or NP components	Peptide sequence modification (humanization), PEGylation/stealth coatings, immunosuppressive coatings/co-delivery, careful selection of NP materials (e.g., natural proteins like ferritin)	[13]
Scalability/cost/regulatory	Complex synthesis, reproducibility, high production cost, regulatory hurdles	Simplified designs, robust self-assembly methods, scalable manufacturing techniques (e.g., microfluidics), cost-effective peptide synthesis, clearer regulatory pathways	[1]
Clinical translation	Poor correlation between preclinical and clinical outcomes	Development of more predictive in vivo models (e.g., PDX, humanized mice), better understanding of NP–biology interactions, improved trial design, theranostic monitoring	[78]

### 5.3. Future Outlook and Perspectives

The field of peptide-based nanoparticles for cancer therapy is poised for continued growth and innovation, driven by advancements in peptide chemistry, materials science, nanotechnology, and cancer biology. Several key trends and future directions are emerging.

There is a strong push towards developing more sophisticated nanoparticles that can sense specific cues within the complex TME—such as altered pH, redox potential, hypoxia, or the overexpression of specific enzymes—and respond by triggering drug release, activating therapeutic moieties, changing morphology to enhance penetration, or switching on imaging signals. In redox-responsive delivery, glutathione (GSH)-responsive paclitaxel (PTX) prodrug micelles, utilizing L-glutathione oxidized (GSSG) as an endogenous linker, have shown efficacy by inducing apoptosis in MCF-7 breast cancer cells [82]. Additionally, disulfide-crosslinked micellar systems have effectively released Doxorubicin (DOX) and nucleic acids in models like HeLa tumor-bearing mice and drug-resistant MCF-7/ADR cells [83]. In enzymatic-responsive systems, Matrix Metalloproteinases (MMPs) and Cathepsin B are key targets [84,85]. MMP-2/9-cleavable oligopeptides, such as GPVGLIGK-NH2, have been incorporated into micelles to deliver Docetaxel (DTX) and α-tocopherol succinate (α-TOS) to solid tumors, enhancing accumulation and reducing systemic toxicity. Cathepsin B-responsive peptides, including Gly–Phe–Leu–Gly–Cys (GFLGC), facilitate the precise release of payloads like DTX from gold nanoparticles, targeting ovarian, breast, and prostate cancers. Another example is the Gly–Phe–Leu–Gly (GFLG) tetrapeptide, used in Cathepsin B-cleavable Doxorubicin (DOX) prodrugs for various solid tumors and hematological malignancies. These intelligent platforms ensure drug activation precisely where needed. External stimuli like light, ultrasound, or magnetic fields are also being explored for spatiotemporal control over nanoparticle activity. This move towards “smart” systems aims to maximize on-target effects while minimizing off-target exposure. Additionally, strategies mimicking natural biological systems, such as coating nanoparticles with cell membranes (e.g., red blood cells, cancer cells) to improve biocompatibility, prolong circulation, and potentially enhance targeting [46], or using natural protein cages like ferritin as carriers [28], are gaining traction. Recognizing that cancer is a complex disease often requiring multimodal treatment, future strategies will increasingly focus on using peptide nanoparticles to facilitate combination therapies. This involves co-delivering multiple therapeutic agents (e.g., chemotherapy + immunotherapy, drug + gene therapy, photosensitizer + drug) within a single nanoparticle platform to achieve synergistic effects, overcome resistance mechanisms, and target multiple pathways simultaneously [48].

Addressing tumor heterogeneity requires personalized approaches [5]. Peptide nanoparticles can be tailored based on the molecular profile of an individual patient’s tumor, for instance, by selecting targeting peptides based on receptor expression or using patient-specific neoantigens for peptide vaccines [73]. Theranostic nanoparticles, which combine diagnostic imaging capabilities (e.g., using peptide-targeted contrast agents or intrinsically active components like iron oxide or fluorescent peptides) with therapeutic functions, hold great promise for the real-time monitoring of drug delivery, assessing treatment response, and guiding personalized treatment decisions. Continued progress in peptide science will yield novel peptides with improved properties. This includes the development of stapled peptides for enhanced stability and cell permeability, the further optimization of cyclic peptides for affinity and resistance, the incorporation of unnatural amino acids to resist proteolysis, and the design of multifunctional peptides integrating targeting, penetration, and therapeutic activities [47]. Computational methods, machine learning, and high-throughput screening platforms will accelerate the discovery and optimization of peptide sequences for specific functions. Furthermore, the integration of artificial intelligence (AI) and machine learning (ML) approaches represents a rapidly advancing frontier poised to significantly contribute to this acceleration, particularly in the design and optimization of peptide-based nanoparticles [86]. These computational tools are increasingly being employed for the de novo design of peptides with desired therapeutic activities, enhanced stability, or specific self-assembling properties. AI/ML algorithms can analyze vast datasets of peptide sequences and experimental outcomes to predict how novel peptides might behave, including their propensity to form specific nanostructures, their ability to bind to particular cancer cell targets, or their potential immunogenicity [87]. By enabling high-throughput virtual screening and rational design, these in silico methods can streamline the development pipeline, reduce experimental costs, and uncover novel peptide candidates for nanoparticle-based cancer therapy that might be missed by traditional discovery approaches [88]. Eradicating minimal residual disease and preventing metastasis are major unmet needs in oncology. Future research will likely focus more on developing peptide nanoparticles specifically designed to target cancer stem cells (CSCs), which drive recurrence and resistance [54], or to interfere with the metastatic cascade by targeting circulating tumor cells or pre-metastatic niches [64].

In conclusion, peptide-based nanoparticles stand at the confluence of materials science, peptide chemistry, and oncology, offering a highly versatile and potent platform for developing next-generation cancer therapies. While significant challenges related to in vivo delivery, clinical translation, and manufacturing remain, the rapid pace of innovation in peptide design, nanoparticle engineering, and understanding of tumor biology suggests a bright future. Continued interdisciplinary collaboration will be essential to bridge the gap between promising laboratory discoveries and impactful clinical applications, ultimately realizing the transformative potential of peptide nanoparticles for improving patient outcomes in the fight against cancer.
